# Investigation of the rate-mediated form-function relationship in biological puncture

**DOI:** 10.1038/s41598-023-39092-8

**Published:** 2023-07-26

**Authors:** Bingyang Zhang, Philip S. L. Anderson

**Affiliations:** Department of Evolution, Ecology, and Behavior, School of Integrative Biology, University of Illinois Urbana-Champaign, Urbana, IL 61801 USA

**Keywords:** Biomechanics, Mechanical engineering, Soft materials, Evolution

## Abstract

Puncture is a vital mechanism for survival in a wide range of organisms across phyla, serving biological functions such as prey capture, defense, and reproduction. Understanding how the shape of the puncture tool affects its functional performance is crucial to uncovering the mechanics underlying the diversity and evolution of puncture-based systems. However, such form-function relationships are often complicated by the dynamic nature of living systems. Puncture systems in particular operate over a wide range of speeds to penetrate biological tissues. Current studies on puncture biomechanics lack systematic characterization of the complex, rate-mediated, interaction between tool and material across this dynamic range. To fill this knowledge gap, we establish a highly controlled experimental framework for dynamic puncture to investigate the relationship between the puncture performance (characterized by the depth of puncture) and the tool sharpness (characterized by the cusp angle) across a wide range of bio-relevant puncture speeds (from quasi-static to $\sim$ 50 m/s). Our results show that the sensitivity of puncture performance to variations in tool sharpness reduces at higher puncture speeds. This trend is likely due to rate-based viscoelastic and inertial effects arising from how materials respond to dynamic loads. The rate-dependent form-function relationship has important biological implications: While passive/low-speed puncture organisms likely rely heavily on sharp puncture tools to successfully penetrate and maintain functionalities, higher-speed puncture systems may allow for greater variability in puncture tool shape due to the relatively geometric-insensitive puncture performance, allowing for higher adaptability during the evolutionary process to other mechanical factors.

## Introduction

Identifying the influence of morphology on functional performance is essential to understanding the evolution of biomechanical systems. However, the relationship between form and function is often complicated by both inherent features of the system as well as external factors that can exert a heavy influence on performance^1^. The inherent complexity of multi-part biomechanical systems often leads to non-linear relationships between form and function^2,3^, while external factors such as temperature can highly alter the performance of physiological systems^4–6^. One factor that can exert a strong influence on form-function relationships across a wide range of biology is the dynamics of the system^1,7–10^. An example of this with far-reaching consequences is that the rate at which a biological material is loaded (strain rate) can influence its response to said load, with materials often becoming stiffer or tougher at higher strain rates^1,11–13^. This strain rate dependence in biomaterials may have a large effect on their resistance to damage, which in turn could greatly affect the performance of a system. Here, we explore how strain rate potentially alters the form-function relationship for a specific type of damage: biological puncture.

**Figure 1:**
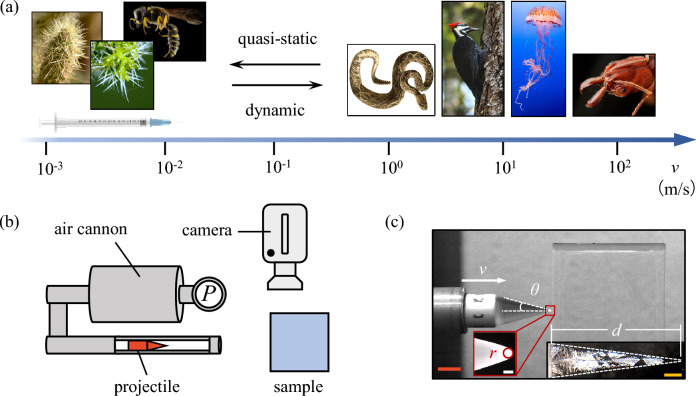
Dynamic puncture. (**a**) The operating speeds of common biological puncture systems (from left to right: jumping cholla (*Opuntia fulgida*; photo credit: San Bernardino National Forest, source: flickr.com), stinging nettle (photo credit: vatra voda, source: unsplash.com), hornet (photo credit: the United States Geological Survey, source: unsplash.com), viper (*Crotalus adamanteus*; photo credit: The New York Public Library, source: unsplash.com), pileated woodpecker (photo by Jasper Garratt, source: unsplash.com), jellyfish (photo credit: Ganapathy Kumar, Monterey Bay Aquarium, source: unsplash.com), trap-jaw ant (Formicidae, *Odontomachus clarus*; adapted from photo by Jen Schlauch, source: flickr.com) and an artificial puncture tool (medical needle) span six orders of magnitude. They can be categorized into quasi-static and dynamic regimes. (**b**) A schematic illustration (top view) of the setups of the dynamic puncture experiment. (**c**) A still-frame high-speed image of the puncturing projectile captured at the onset of impact with the material sample. The velocity (*v*) and half cusp angle ($\theta$) of the projectile are determined using image processing. Inset left: microscopic image of the tip of the projectile, where the tip radius (*r*) is measured; inset right: representative microscopic image of an undeformed puncture fracture surface produced by a projectile with $2\theta =30^{\circ }$ at $v\approx 50$ m/s. White dashed lines are included to outline the fracture surface. Scale bars: 10 mm (orange); 200 μm (white); 4 mm (yellow).

Biological puncture systems appear, on the surface, to show a straightforward relationship between morphology and functional performance^14^. Tool sharpness has been shown to significantly alter puncture performance in a range of organisms^15–18^. However, for the most part, these studies have only tested the effect of sharpness at quasi-static/low speeds ($< 1{\mathrm{m/s}}$), while biological puncture events can occur upwards of 60 m/s or more^1,19–21^ (Fig. 1). Work on dynamic puncture events (> 1 m/s) has shown that at high speeds strain-stiffening occurs in target materials, particularly when the material is soft and deformable^22–24^. Given the shift in material response at high rates of loading, does the relationship between tool shape and puncture performance change at higher puncture speeds?

We set out here to test this idea across a range of biologically relevant puncture speeds: from passive puncture systems (e.g. cactus spines, rose prickles) to dynamic puncture systems such as snake strikes at $\approx$ 3 m/s^25,26^, woodpeckers at $\approx$ 7 m/s^27^, and cone snails at $\approx$ 19 m/s^28^ up to jellyfish stinging cells at $\approx$ 30 m/s^29^ and trap-jaw ants at $\approx$ 60 m/s^19^. The central hypothesis we test is whether the relationship between puncture tool sharpness (quantified as cusp angle) and puncture performance (measured as depth of puncture) will remain constant across puncture speeds when penetrating soft, stretchable tissues. While the constancy of the relationship between tool shape and puncture performance at all speeds is an implicit assumption in many studies, we speculate that altering the strain rate of the puncture event will potentially change the substrate material’s apparent toughness, due to both the inertial and the viscoelastic effects at the micro-molecular scale. If the relationship between tool form and puncture performance changes at different speeds, it will have significant consequences for the ecology and evolution of these puncture systems.

## Results

**Figure 2:**
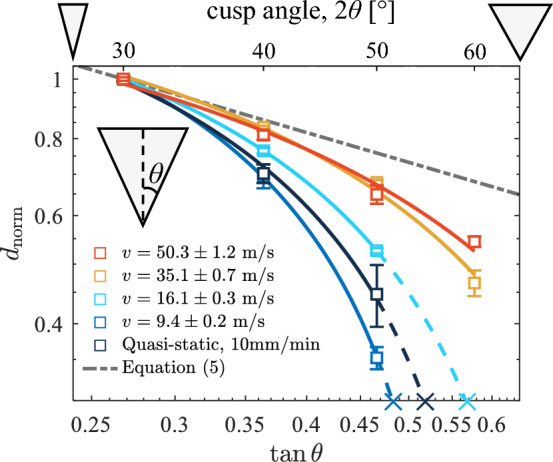
Rate-dependent angular sensitivity: the dependence of the normalized depth of puncture ($d_{\text {norm}}$) on cusp angle ($2\theta$) variations at different puncture speeds (*v*). As *v* increases from a quasi-static condition (10 mm/min) to $v\approx 50$ m/s, the sensitivity of $d_{\text {norm}}$ to changes of $\theta$ diminishes. The slope of the fitting curve approaches that of the estimated upper bound, i.e., (5). Note puncture is not possible for $2\theta =60^{\circ }$ at the three lowest puncture speeds (crosses). The dashed lines indicate extrapolations of the fitting curves. The coefficients of determination of the curve fits: $R^2 \ge 0.99$. Controlled tip radius: $r \approx 110 \pm 12$ μm.

To quantify the dependence of puncture performance on the cusp angle of the puncture tool and determine how variations in puncture speed influence the sensitivity of such dependence, we measure depth of puncture (*d*) as a function of cusp angle ($2\theta =[30^{\circ }, 40^{\circ }, 50^{\circ }, 60^{\circ }]$) under a controlled tip radius ($r \approx 110 \pm 12$ μm) and five different loading rates. Figure 2 illustrates the results on a log-log scale. The five different puncture speeds (*v*) include one quasi-static condition (loading rate: 10 mm/min, deep blue) and four dynamic conditions: $v=9.4\pm 0.2~\text {m/s}$ (light blue), $v=16.1\pm 0.3~\text {m/s}$ (teal), $v=35.1\pm 0.7~\text {m/s}$ (light orange), and $v=50.3\pm 1.2~\text {m/s}$ (orange). While the magnitude of *d* for a puncture tool increases monotonically with speed due to the increased kinetic energy, in Fig. 2 the resultant *d* values within the same test speed condition are normalized by the maximum measurement across the tested range of angles (i.e., $d_{\text {norm}}=d/d_{\max }$, where $d_{\max }$ occurs at the smallest tested angle ($30^{\circ }$)) to isolate the angular-dependent trend from the magnitude effect. Each error-barred data point represents the average and standard deviation obtained from three individual tests.

To capture the slopes of the trends in Fig. 2 as an evaluation for the sensitivity of the $d_{\text {norm}}$ values to $\theta$ changes, a power-law function is selected to fit the data points at each tested speed

1 $$\begin{aligned} d_{\text {norm}}=d_{\text {norm,0}}+k (\tan \theta )^n, \end{aligned}$$

where $d_{\text {norm,0}}$ represents the depth of puncture at the limit $\theta \rightarrow 0$, and *k* and *n* are fitting parameters. The resultant power-law fitting curves are highlighted on a log-log scale in Fig. 2, and the corresponding exponent values (*n*) are listed in Table 1. The trends observed in Fig. 2 offer several insights into the effects of load rate on the relationship between cusp angle and puncture depth: (1) The $d_{\text {norm}}$ values decrease with increasing $\theta$ within the range of measurement ($2\theta =30^{\circ }-60^{\circ }$); (2) The slope of the fitting curve significantly reduces as the puncture speed increases. This diminishing sensitivity is evident from the resultant exponent values (Table 1); (3) For a tool with controlled tip radius ($r \approx 110 \pm 12$ μm) and initial energy investment, and $2\theta =60^{\circ }$, successful puncture was only achieved at relatively high speeds (i.e., $v=35.1\pm 0.7~\text {m/s}$ and $v=50.3\pm 1.2~\text {m/s}$); (4) The sensitivity at the lowest test speed (i.e., $v=9.4\pm 0.2~\text {m/s}$) is similar to that obtained at the quasi-static test condition, while the latter exhibits a slightly larger exponent value (Table 1). These observations imply a significant rate-dependent geometric effect on the puncture performance associated with cusp angle variations.

**Table 1:** Exponent values from fitting curves in Fig. 2.

Speed (m/s)	Quasi-static	$9.4\pm 0.2$	$16.1\pm 0.3$	$35.1\pm 0.7$	$50.3\pm 1.2$
Exponent	0.94	1.07	0.84	0.67	0.58

## Discussion

### Rate-dependent angular sensitivity

The positively correlated form-function relationship between the sharpness of puncture tools (measured by height-to-base aspect ratio, cusp angle, or tip radius) and puncture performance (characterized by the force or energy required for puncture) has been experimentally demonstrated by numerous studies on biological puncture^15–18,30–32^. Therefore, it is not surprising that Fig. 2 exhibits overall decreasing trends of $d_{\text {norm}}$, indicating a diminishing puncture performance with increasing $2\theta$ values (i.e., decreasing sharpness/slenderness) across a wide range of tested speeds. Underlying this dependence are the changes in the transfer and distribution of the local energy with respect to the $2\theta$ variations, as demonstrated theoretically by our previously established puncture energy model^24^. The initial energy investment/loss of kinetic energy due to puncture, $\Delta E_{k}$, is divided into three energy contributions, i.e.

2 $$\begin{aligned} \Delta E_k=W+\Delta U_{\text {el}}+W_f, \end{aligned}$$

where *W* is the energy/work done to create fracture surface, $\Delta U_{\text {el}}$ is the energy contribution from elastic deformation required to accommodate the penetrating tool, and $W_f$ is the dissipated energy due to friction as the tool moves through the material. In a soft hyperelastic material substrate, for example, more elastic energy contribution ($\Delta U_{\text {el}}$) is required for a larger $2\theta$ value to displace the material to accommodate a larger tool radial size. The energy contributions from *W* and $W_f$ also increase monotonically with $2\theta$, though they do so at a lower rate than $\Delta U_{\text {el}}$^24^. As a result, for the same initial energy investment (i.e., a controlled initial kinetic energy), the total depth of puncture, *d*, reduces as the puncture tool widens. Additionally, we note that variations in tip radius can also influence the puncture performance (i.e., the magnitude of $d_{\text {norm}}$, see Supplementary Fig. S1). However, our previous model^24^ has demonstrated that for a sharp tool, the energy effect associated with the radius is minor compared to that associated with the cusp angle. This is further supported by our results in Supplementary Information where the radius vs. depth of puncture relationship exhibits very limited rate dependence (Supplementary Fig. S1). For this reason, we focus on the angular effect for the rest of the discussion where the tool tip radius is controlled.

To describe the reduced sensitivity of $d_{\text {norm}}$ to $2\theta$ variations at larger speeds as observed in Fig. 2, rate dependence needs to be included in the puncture energy model (2). We hypothesize that such rate dependence originates from the effects of viscoelastic dissipation and inertia — both are no longer negligible at higher puncture speeds. Consequently, the fracture toughness (*G*) of the tested material substrate increases, which ultimately results in a higher magnitude of *W* and a reduced angular sensitivity.

Both experimental evidence^33–35^ and linear viscoelastic approximation^36–40^ have demonstrated the rate-dependence of the apparent fracture toughness/critical strain energy release rate (*G*) of soft rubbery materials. *G* takes a general form, at a constant reference temperature, *T*

3 $$\begin{aligned} G=G_0[1+f(v,T)]\sim G_0\left( \frac{v}{v_0}\right) ^{\alpha }\sim G_0\left( \frac{\tau _0}{\tau }\right) ^{\alpha }, \end{aligned}$$

where *f*(*v*, *T*) is a function of the crack velocity (*v*) and *T*, $v_0$ is a reference velocity associated with the characteristic relaxation time scale $\tau _0$ of the material, $\tau$ is the local time scale, and $\alpha$ is a material constant. (3) suggests a significant increase in *G* when the time scale associated with puncture fracture propagation satisfies $\tau \ll \tau _0$. We estimate $\tau \sim 10^{-5}$ s in our puncture tests with higher speeds (assuming a nominal crack tip length scale $\bar{r}\sim 10^{-4}$ m as imposed by the tool tip radius and a nominal crack velocity, $\bar{v}\sim 10~\mathrm {m/s}$). For comparison, a silicone rubber (Sylgard 184, 20:1 mixing ratio) having similar properties to our model material^41^ has an approximate relaxation time, $\tau _0 \sim 0.1$ s^42^.

It is theorized that the inertial effect becomes a significant contributor to *G* when the magnitude of applied *v* approaches or exceeds the speed of sound, *c*, i.e., the speed with which longitudinal stress waves transmit tensile and compressive stresses inside the substrate^40^. We note that this scenario may occur for our three highest puncture speeds ($v \approx 16~\mathrm {m/s}$, $35~\mathrm {m/s}$ and $50~\mathrm {m/s}$): the longitudinal stress wave speed of our model silicone material satisfies the approximation, $c=\sqrt{E/\rho } \approx 20~\mathrm {m/s}$^43^, where the elastic modulus is measured to be $E \approx 0.39$ MPa^41^, and the density is $\rho \approx 990~\mathrm {kg/m^3}$^44^.

The above order-of-magnitude estimations suggest a larger *G* value at a higher puncture speed. It can be shown from an expanded form^24^ of (2) that this will theoretically lead to a smaller decrease of the $d_{\text {norm}}$ value and thus a lower angular sensitivity for the same constitutive properties and increment of $2\theta$. In an extreme case where the increase of *G* is so large such that the magnitude of *W* dominates over those of $\Delta U_{\text {el}}$ and $W_f$, we have the approximation^24^

4 $$\begin{aligned} \Delta E_k \approx W \sim f(\lambda _m)\tan \theta G d^2, \end{aligned}$$

where the second part of (4) assumes that the puncture produces a triangular planar crack in the undeformed configuration; and $f(\lambda _m)$ denotes a dimensionless function of limiting stretch $\lambda _m$ in tension. Solving for *d* from (4) leads to an estimation of the magnitude of $d_{\text {norm}}$ as a function of $\tan \theta$ when $G \gg \mu d$ (where $\mu$ is the shear modulus)

5 $$\begin{aligned} d_{\text {norm}} \sim \left( \frac{\tan \theta _0}{\tan \theta }\right) ^{\frac{1}{2}}, \end{aligned}$$

where $\theta _0=15^{\circ }$ is chosen to be the reference half angle of the tool for the normalization. Essentially, (5) estimates the upper limit of $d_{\text {norm}}$ given $\theta$. We include it in Fig. 2 to further compare with the experimental measurements. As expected, the fit curves approach the theoretical limit as the speed increases within our tested range.

Some interesting observations noted in the “Results” section remain to be further investigated. The incapability of penetration occurring for tools with $2\theta =60^{\circ }$ at lower speeds may be attributed to the significantly increased force necessary for fracture initiation, as evident from the quasi-static measurements. However, we note that the maximum *d* value we characterize is associated with fracture propagation. It is unclear why the angle-dependent slope calculated at $v=9.4\pm 0.2~\text {m/s}$ is slightly lower than that from the quasi-static tests in Fig. 2. While the two test conditions may both have a minor rate-dependent effect, perhaps the instantaneous crack speed associated with the sudden release of energy at the onset of the crack initiation during the quasi-static puncture is large enough to cause a slight increase of the $d_{\text {norm}}$ value at large $\theta$. Given these speculations, the initiation of fracture associated with puncture is a vital avenue for future exploration.

### Implications for biological puncture systems

The rate-dependent angular sensitivity discussed above may have far-reaching implications for the form-function relationship underlying the evolution of biological puncture systems. The range of loading rates used for our experiments covers the majority of puncture speeds reported across biological systems. At the low end, the quasi-static tests ($\lesssim 1$ mm/s) are similar to the loading rates of either passive puncture tools, such as cactus spines^45^, stinging nettle^46^, and porcupine quills^47^, or low-speed active puncture systems such as wasp stingers^48^. The range of speeds used in the dynamic puncture tests ($\sim$ 9–50 m/s) shares the same orders of magnitude with the average operating speeds of most of the actively-puncturing living organisms reported in the literature, from snake strikes ($\approx 3$ m/s)^25,26^ to cnidarian nematocytes ($\approx 30$ m/s)^29^ to trap-jaw ants ($\approx 60$ m/s)^19^. The decreased sensitivity of $d_{\text {norm}}$ to sharpness measures as speed increases (Fig. 2) offers several insights into how the morphology-performance relationship plays out across the dynamic range of biological systems: 1) Passive/low-speed puncture systems are likely highly reliant on sufficiently sharp puncture tools to achieve efficient penetration of soft tissues; 2) Dynamic puncture systems with high loading rates can hypothetically sustain a relatively higher degree of morphological variation without trading off too much of their maximum puncture performance.

In passive/low-speed systems, puncture performance (characterized by force, energy, or damage size) will be more dependent on the shape of the puncture tool (e.g., Fig. 2). Evidence for such dependence has been shown in experiments on viper fang^18^, canine teeth^16^, and bio-inspired needles^49^ during quasi-static puncture experiments. Active puncture systems operating at quasi-static load rates, like these examples, can still overcome having blunter tools by simply increasing the force with which they puncture. However, passive puncturing systems (e.g. cactus spines, rose prickles) do not gain energy from the puncturing organism, but from the target brushing against them. We speculate that such a mechanical restriction demands higher levels of sharpness in their puncture tools to not only maintain a level of performance (puncture depth), but to ensure successful puncture occurs at all. A sufficiently sharp/slender puncture tool becomes essential for their biological functionalities. For example, jumping cholla (cactus) relies on the successful puncture with its sharp spines to attach its terminal cladodes to passing animals for dispersal^14,45^; Stinging nettles’ defensive mechanism via neurotoxin injection depends on successful penetration of mammalian skins with their sharp stinging trichomes^46^. At the same time, successful puncture of passive systems requires sufficient strength and stiffness of the puncture tool as well, especially for the non-sacrificial ones. This poses a potential trade-off between the tool being of sufficient sharpness/slenderness and still robust enough to penetrate the target without deflecting or breaking. The combination of these effects in passive systems implies that there is potentially a mechanical limitation on morphological variability, resulting in a potentially true mechanical constraint on their evolution.

In contrast, dynamic, high-speed puncture systems may achieve a level of mechanical release on their diversification potential. Higher variability in the puncture tool shape stemming from the relatively low level of geometric sensitivity of the puncture performance at higher speeds (Fig. 2) provides a ‘degree of freedom’ for the evolutionary process by releasing on one axis of variation. This variability allows these puncture tools more freedom to evolve for other mechanical factors that may negatively impact the puncture performance than in quasi-static systems. For example, a larger cusp angle can result in higher structural integrity against buckling or fracture^50^ and therefore, release dynamic systems from the constraints of strength and stiffness, while a passive system may need to develop a non-conical shape having a wider base to achieve a similar effect while maintaining puncture performance^51^. Organisms that puncture under water (e.g., spearing mantis shrimp strikes^52^ and shark teeth bites^53^) could adapt a unique puncture tool shape for concurrent optimization of the puncture and hydrodynamic performance.

Overall, active, high-speed biological puncture tools may not need to be subjected to as much developmental control as passive systems do, allowing for greater epigenetic variation as well. This, coupled with the ability to adapt to other mechanical pressures, suggests that active, dynamic puncture systems should show higher levels of both intraspecific and interspecific variation in tool form than passive puncture systems. In an initial survey of the literature, we found preliminary evidence for the above-proposed relationship between shape variations (tool cusp angle) and operating speeds in various biological puncture systems. All cusp angles reported here were either taken from reported measurements or our own estimates based on the figures presented in the papers. In a passive puncture system such as cactus spines, we estimated from scanning electron microscope (SEM) images of the spines from six species^45^ that the cusp angle of the conical body of the spines (which is responsible for the fracture propagation process discussed in this work) lies in a narrow range of $7.7^{\circ } \pm 1.8^{\circ }$. The cusp angle of the tapered region of the prickles/spines collected from three species of climbing plants and a cultivar^51^ was estimated to vary within a range of $12.4^{\circ } \pm 2.0^{\circ }$. In contrast, the dynamic puncture systems we found examples of in the literature seem to exhibit a higher degree of tool shape diversity. Viper fangs, for example, which puncture targets at $\sim 3$ m/s^25,26^, exhibit reported cusp angles in the literature ranging between $\sim 25^{\circ }$ and $\sim 45^{\circ }$ across 19 species, with a cluster of data around $30^{\circ }$^18^. Shark teeth show a range of variations of $\sim 10$–$40^{\circ }$ in their cusp angles in the Family Lamnidae across three genera^54^. For context, it has been reported that white sharks have a burst speed of $\sim 11$ m/s when approaching a target^55^. We speculate the speed of puncture of the teeth can be even higher when the motion of the jaw is taken into account. Fish-hunting cone snails strike and spear their prey at an average peak velocity $\sim 19$ m/s^28^. SEM images of their radular harpoon^56^ allowed us to estimate the average tip cusp angle across eight species: $24.5^{\circ } \pm 6.6^{\circ }$. In faster puncture systems such as the stinging organelles (nematocysts) of cnidarians and dinoflagellates^57^, the barb/stylet structure penetrates the target within microseconds at a final velocity of $\sim 30$ m/s^29^. These microscopic puncture tools exhibit diverse tapered shapes. Their variations are not only interspecific but also appear in individual puncture tools due to widening when moving away from the tip. We roughly estimated the cusp angles range from $\sim 15$ to $\sim 50^{\circ }$ from the nematocysts of four species^57–61^. Overall, the above initial findings verify the biological relevance of our proposed experimental framework in terms of both puncture tool shape and rate. Although this survey presents very preliminary evidence, it takes an initial step toward further systematic comparative analysis necessary to quantify the spread and diversity of the puncture tool shape in relation to the puncture speed across different phyla. Such future studies should also examine additional factors such as the puncture tool curvature and the material the tool is composed of. Nonetheless, we hope our framework can shed further light on the complexity of biological puncture systems and fuel further discussion and exploration of the interplay between the effects of rate and morphology in puncture systems.

### Conclusion

Organisms across different phyla achieve successful puncture under a range of operating speeds spanning six orders of magnitude. Underlying their puncture mechanisms is the complex interaction between puncture tool geometries, material-mediated puncture performance, and rate dependence. Our experimental work with a highly controlled dynamic puncture system demonstrates that puncture speed, within a wide bio-relevant range, has a significant impact on the geometry dependence of puncture performance: At a quasi-static condition or relatively low speeds, the depth of puncture serving as a performance indicator decreases rapidly with the cusp angle of the puncture projectile; However, the slope of the depth of puncture vs. angle relationship diminishes as puncture speed increases. This rate-dependent angular sensitivity has strong implications for the evolutionary processes of both slow and fast biological puncture systems, as the mechanical constraints imposed on the former are partly lifted for the latter by releasing on one axis of geometric variation. These results provide solid evidence and an experimental basis from which a future biomechanical framework can be established to explore how physical principles shape the evolution of highly disparate biological puncture systems undertaking complex, dynamic puncture strategies.

## Methods

### Silicone elastomer samples

A silicone elastomer (Solaris, Smooth-On, Inc.) is selected and fabricated as the model material for puncture experiments following documented procedures^41^: Part A and part B prepolymer kits as received are mixed in a 1:1 weight ratio. The liquid mixer is degassed for approximately 30 minutes before being transferred into a standard plastic cube mold (ASTM C-109). The inner walls of the mold are coated with 0.02-inch thin polycarbonate sheets to produce smooth sample surfaces and ensure transparency of the sample. Sample cubes (dimensions: $\approx$ 49 mm $\times$ 49 mm $\times$ 42 mm, $\text {thickness}\times \text {width}\times \text {height}$; weight: $\approx 100 \pm 1$ g) are obtained after curing and setting at room temperature for approximately 48 hours.

### Dynamic puncture method

Dynamic puncture tests are performed using a customized compressed air cannon (Fig. 1b) (Ballistic Loading and Structural Testing Lab (BLAST), NC State University). Projectiles for testing are manufactured using a stereolithography (SLA) 3D printer (Form 3, Formlabs Inc., clear resin, FLGPCL04). Each projectile consists of a cylindrical base that transitions to a conical section (Fig. 1c) having a controlled cusp angle ($2\theta =[30^{\circ }, 40^{\circ }, 50^{\circ }, 60^{\circ }]$) and tip radius (Fig. 1c and Supplementary Fig. S1). Two projectile lengths ($\approx$ 35 and 73 mm) are selected to accommodate variations in the depth of puncture at different testing speeds. The tip of the projectile as manufactured is sharpened or dulled using sandpaper (2000 grit) and cleaned thoroughly before testing. The prepared tips are examined under a stereo microscope (Fig. 1c) (M205C, Leica Microsystems Inc.) before and after a set of puncture tests to measure the radius and examine structural integrity, respectively. During a puncture test, a projectile is loaded into the barrel of the cannon at a designed position (Fig. 1b) before being propelled out by a calibrated pressure (ranging from 10 psi to 50 psi) to impact a target sample. The sample is positioned at a distance such that the onset of impact occurs immediately after the projectile has fully emerged from the barrel. Therefore, the muzzle velocity is maintained and the speed at the onset of impact is controlled. The puncture speed (as listed in Table 1) is calibrated via a high-speed camera (FASTCAM SA-Z, Photron Inc.) and post-image and video processing (ImageJ). The large difference between the weight of the sample ($\approx 100$ g) and the weight of the projectile ($\approx 3.6$ g for shorter ones and $\approx 5.2$ g for longer ones) by design, combined with the adhesion between the sample and the supporting stage, ensures that the projectile comes to nearly a full stop at the maximum depth of puncture and the system becomes momentarily stationary before the projectile and the sample are mutually repelled by the released elastic deformation.

### Quasi-static puncture method

Quasi-static puncture tests are carried out using a universal test stand (Instron 5944, Instron Inc.) (Fig. S2). A 3D-printed projectile as described in the dynamic puncture method (length: $\approx$ 73 mm; $2\theta =[30^{\circ }, 40^{\circ }, 50^{\circ }, 60^{\circ }]$) is mounted to the crosshead before being descended and inserted into a silicone sample cube placed on a compression stage at a feeding speed of 10 mm/min. The full force-displacement response is measured during the puncture process to 1) detect the onset of fracture initiation; 2) calculate the area under the curve as an estimation of the total work done/energy investment. For each selected cusp angle, a controlled total work done ($0.26\pm 0.01$ J) is applied by controlling the maximum displacement. The magnitude of the work done is calibrated such that the maximum displacement is larger than the critical displacement at the onset of crack initiation for at least $2\theta \le 50^{\circ }$; At the same time, the maximum depth of puncture in the undeformed configuration is smaller than the available length of the conical region of the puncture tool.

### Characterization of the puncture fracture

Microscopic images of the undeformed puncture fracture surface following the removal of the puncture tool from the material substrate are captured using a stereo microscope (M205C, Leica Microsystems Inc.). The images are stitched together in post-processing if necessary to reconstruct the full side view of the fracture surface (e.g., Fig. 1c) using ImageJ. The depth of puncture is determined from both microscopic image processing and manual probing (parallel probe insertion adjacent to the fracture surface) by measuring and averaging the distance between the vertex of the fracture surface and the superficial crack opening along the centerline.

## Supplementary Information

Supplementary Information.

## Data Availability

All data generated or analyzed during this study are included in this manuscript and its supplementary information.
