# Supplementary material for: Investigation of the rate-mediated form-function relationship in biological puncture
Source: Sci Rep. 2023 Jul 26;13:12097. doi: 10.1038/s41598-023-39092-8 (PMC10372153; doi:10.1038/s41598-023-39092-8)
Supplement: Supplementary file 1 — Supplementary Information. [file 41598_2023_39092_MOESM1_ESM.pdf]

Supplementary Information: Investigation of the rate-mediated form-function relationship in biological puncture

Bingyang Zhang[1,*] **and Philip S L Anderson**[1]

[1]Department of Evolution, Ecology, and Behavior, School of Integrative Biology, University of Illinois Urbana-Champaign, Urbana, IL 61801, USA.
[*]bzhang53@illinois.edu

Effect of tip radius

The effect of variations in the puncture tool tip radius (r) on the dynamic puncture performance (characterized by the normalized depth of puncture d_{norm}) is evaluated following a method similar to that described in the main text for the angle effect to examine possible divergence from the results in Fig. 2 (main text). Fig. S1 plots the d_{norm} values as a function of r (average selected radii: $r \approx 36\ \mu$m, $r \approx 107\ \mu$m, and $r \approx 416\ \mu$m; controlled cusp angle: $2\theta = 30^{\circ}$) measured at three different puncture speeds (quasi-static puncture test: loading rate: 10 mm/min (deep blue); $v = 9.6 \pm 0.3$ m/s (light blue); and $v = 34.0 \pm 0.6$ m/s (yellow)). Each data point represents the average value calculated from at least two individual tests. The vertical and horizontal error bars indicate the standard deviations of d_{norm} and r, respectively. Fig. S1 shows an apparent discrepancy between the effect of angle and the effect of radius on d_{norm}: While the d_{norm} values also decrease at larger tip radii, the rate dependence of the trend seems to be minor within our range of measurements. However, it should be noted that for the largest tip radius tested ($r \approx 416\ \mu$m), puncture becomes more difficult at a lower testing speed: In more than 50% of the attempted tests at the quasi-static condition, the puncture tool failed to penetrate the material; while puncture was not possible for $v = 9.6 \pm 0.3$ m/s.

Among the three puncture speeds tested in Fig. S1, there is no systematic change in the sensitivity/slope of the radius dependence. This observation is consistent with our previous hypothesis[1]: The elastic and dissipative energy contributions to puncture that are localized at the tip of a sharp puncture tool are minor compared to those associated with the body of the puncture tool. Therefore, for a controlled cusp angle ($2\theta = 30^{\circ}$) and a relatively small tip radius, $r \lesssim 100\ \mu$m, the effect of angle dominates over the effect of tip radius. Consequently, the magnitude of d_{norm} and its sensitivity to the changes of r stay at a relatively constant level as the puncture speed increases. Only at a larger r value ($r \gtrsim 400\ \mu$m) where the radius effect becomes more significant do the measured values of d_{norm} exhibit a small divergence and the geometric sensitivity slightly decreases at $v = 34 \pm 0.6$ m/s. This behavior is presumably related to the expanded large deformation and dissipative fields near the crack tip with a large imposed radius. As a result, more energy is required to sustain the crack propagation, and less energy remains available for the creation of the fracture surface for the same initial energy investment[1], causing the overall decreasing trend of d_{norm}.

Calculations for effective depth of puncture

To enable comparison of large depth of puncture values, d, between different tool shapes at the two highest testing speeds, a special mathematical treatment is applied to some raw measurements of d to calculate the effective depth of puncture, d_{eff}. Such treatment is only necessary when the penetration exceeds the available length of the conical region and extends into the cylindrical region of the puncture tool. In Fig. 2, the four data points corresponding to $v = 50.3 \pm 1.2$ m/s and the two data points corresponding to $v = 35.1 \pm 0.7$ m/s and $2\theta = [40^{\circ}, 50^{\circ}]$ represent effective depth of puncture values. The effective depth of puncture takes a form

$$d_{\mathrm{eff}} = l_{\mathrm{cone}} + l_{\mathrm{eff}} \tag{1}$$

where l_{cone} is the maximum length of the conical region of the tool, and l_{eff} is the effective length of the cone converted from the cylindrical portion of the measured d value, d_{cyl} (*i.e.*, $d = d_{\mathrm{cyl}} + l_{\mathrm{cone}}$). The conversion is established based on the conservation of energy and under the assumption of similar frictional contributions, such that

$$\begin{aligned} E_{\mathrm{conv}} = W_{\mathrm{cyl}}(d_{\mathrm{cyl}}) + \Delta U_{\mathrm{el,cyl}}(d_{\mathrm{cyl}}) = W_{\mathrm{cone}}(d_{\mathrm{eff}}) \\ + \Delta U_{\mathrm{el,cone}}(d_{\mathrm{eff}}) - W_{\mathrm{cone}}(l_{\mathrm{cone}}) - \Delta U_{\mathrm{el,cone}}(l_{\mathrm{cone}}) \end{aligned} \tag{2}$$

where E_{conv} is the converted energy contribution, W is fracture dissipation, and ΔU_{el} is stored elastic energy, with the subscripts 'cyl' and 'cone' indicating the energy associated with a cylindrical and a conical puncture tool, respectively. We refer to References[2] and[1] for the full expressions of the cylindrical and conical puncture energy solutions, respectively. Combining (1) and (2), we can solve for d_{eff} and l_{eff} for each measured d value.

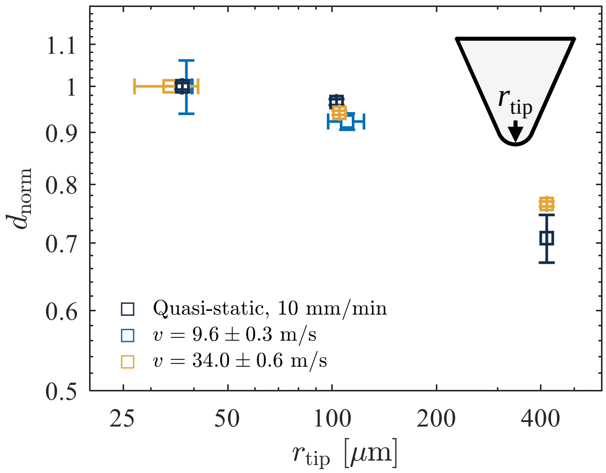

Figure S1. The effect of tip radius (r_{tip}) on the normalized depth of puncture (d_{norm}) at different puncture speeds (v). Within the range of v tested (from a quasi-static condition to $v = 9.6 \pm 0.3$ m/s and $v = 34.0 \pm 0.6$ m/s), the resultant d_{norm} values exhibit similar sensitivity to r_{tip} variations across over one order of magnitude. Note puncture is not possible for the largest tip radius ($r \approx 416$ μm) at $v = 9.6 \pm 0.3$ m/s. Controlled cusp angle: $2\theta = 30°$.

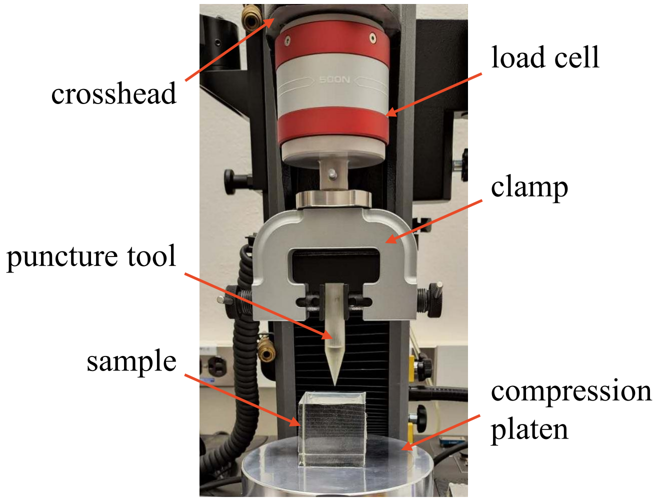

Figure S2. Test instrument for quasi-static puncture tests

References

1. Zhang, B. & Anderson, P. S. L. Modelling biological puncture: a mathematical framework for determining the energetics and scaling. *J. The Royal Soc. Interface* **19**, DOI: 10.1098/rsif.2022.0559 (2022).
2. Shergold, O. A. & Fleck, N. A. Mechanisms of deep penetration of soft solids, with application to the injection and wounding of skin. *Proc. Royal Soc. London. Ser. A: Math. Phys. Eng. Sci.* **460**, 3037–3058, DOI: 10.1098/rspa.2004.1315 (2004).
